# Supplementary material for: Polyketide synthase gene clusters in the ericoid mycorrhizal fungus Oidiodendron maius: genome-wide prediction, expression profiling, and functional characterization of knockout mutants
Source: Front Plant Sci. 2026 Sep 16;17:1924430. doi: 10.3389/fpls.2026.1924430 (PMC13623903; doi:10.3389/fpls.2026.1924430)
Supplement: Supplementary file 1 [file SupplementaryFile1.zip › Supplementary Materials/Supplementary Folder 1.zip!/Oidma1_AssemblyScaffolds_Repeatmasked/knownclusterblast/cluster8/jgi.p_Oidma1_111398_mibig_hits.html]

| MIBiG Protein | Description | MIBiG Cluster | MiBiG Product | % ID | % Coverage | BLAST Score | E-value |
| --- | --- | --- | --- | --- | --- | --- | --- |
| CAI94705.1 | putative\_acid\_AMP\_ligase | BGC0000141 | Rubradirin\_biosynthetic\_gene\_cluster | 28 | 44.3379790941 | 133 | 1.3e-30 |
| KGO40476.1 | AMP-dependent\_synthetase/ligase | BGC0001205 | Communesin\_biosynthetic\_gene\_cluster | 28 | 35.8013937282 | 114 | 6.1e-25 |
| AFV52199.1 | acyl-ACP\_synthetase | BGC0000081 | Kedarcidin\_biosynthetic\_gene\_cluster | 26 | 47.9094076655 | 113 | 1.4e-24 |
| AKA54630.1 | AMP\_dependent\_CoA-ligase | BGC0001216 | Splenocin\_biosynthetic\_gene\_cluster | 29 | 33.275261324 | 106 | 2.8e-22 |
| AEI98652.1 | CtcI | BGC0000209 | Chlortetracycline\_biosynthetic\_gene\_cluster | 28 | 38.4146341463 | 89 | 2.1e-17 |
| AAQ84158.1 | PlmJK | BGC0000123 | Phoslactomycin\_B\_biosynthetic\_gene\_cluster | 24 | 45.6445993031 | 89 | 2.7e-17 |
| BAI63284.1 | putative\_peptide\_arylation\_enzyme | BGC0000434 | SW-163\_biosynthetic\_gene\_cluster | 26 | 34.0592334495 | 80 | 1.7e-14 |
| AEA60663.1 | pyoverdine\_synthetase | BGC0000892 | Caryoynencin\_biosynthetic\_gene\_cluster | 25 | 43.031358885 | 79 | 2.8e-14 |
| CAC17498.1 | putative\_AMP-binding\_ligase | BGC0000324 | Coelibactin\_biosynthetic\_gene\_cluster | 24 | 47.6480836237 | 73 | 2e-12 |
| BAC87906.1 | probable\_acinetobactin\_biosynthesis\_protein | BGC0000294 | Acinetobactin\_biosynthetic\_gene\_cluster | 24 | 45.2961672474 | 67 | 1.1e-10 |
| BAG84247.1 | putative\_L-prolyl-AMP\_ligase | BGC0000257 | Prodigiosin\_biosynthetic\_gene\_cluster | 26 | 28.2229965157 | 64 | 1.2e-09 |
| ACR11042.1 | Enterobactin\_synthetase\_component\_E/2,3-dihydroxybenzoate-AMP\_ligase | BGC0000451 | Turnerbactin\_biosynthetic\_gene\_cluster | 25 | 33.7979094077 | 62 | 4.7e-09 |
| AGZ15459.1 | putative\_non-ribosomal\_peptide\_synthetase | BGC0001036 | Polyoxypeptin\_biosynthetic\_gene\_cluster | 24 | 44.3379790941 | 56 | 3.4e-07 |
| AAS92545.1 | SirP | BGC0001044 | Sirodesmin\_biosynthetic\_gene\_cluster | 24 | 34.3205574913 | 52 | 3.7e-06 |
